# Active Tuberculosis Case Finding in Port-au-Prince, Haiti: Experiences, Results, and Implications for Tuberculosis Control Programs

**DOI:** 10.1155/2016/8020745

**Published:** 2016-09-05

**Authors:** Guesly J. Delva, Ingrid Francois, Cassidy W. Claassen, Darwin Dorestan, Barbara Bastien, Sandra Medina-Moreno, Dumesle St. Fort, Robert R. Redfield, Ulrike K. Buchwald

**Affiliations:** ^1^Department of Medicine, Institute of Human Virology, University of Maryland School of Medicine, 725 West Lombard Street, Baltimore, MD 21201, USA; ^2^Division of Infectious Diseases, Department of Medicine, University of Maryland School of Medicine, 725 West Lombard Street, Baltimore, MD 21201, USA; ^3^Institute of Human Virology, University of Maryland School of Medicine, 4, Delmas 81, Port-au-Prince, Haiti

## Abstract

*Background*. Haiti has the highest tuberculosis (TB) prevalence in the Americas with 254 cases per 100,000 persons. Case detection relies on passive detection and TB services in many regions suffer from poor diagnostic and clinical resources.* Methods*.* Mache Chache* (“Go and Seek”) was a TB REACH Wave 3 funded TB case finding project in Port-au-Prince between July 2013 and September 2014, targeting four intervention areas with insufficient TB diagnostic performance.* Results*. Based on a verbal symptom screen emphasizing the presence of cough, the project identified 11,150 (11.75%) of all screened persons as TB subjects and 2.67% as smear-positive (SS+) TB cases. Enhanced case finding and strengthening of laboratory services led to a 59% increase in bacteriologically confirmed cases in the evaluation population. In addition, smear grades dropped significantly, suggesting earlier case detection. Xpert® MTB/RIF was successfully introduced and improved TB diagnosis in HIV-infected, smear-negative clinic patients, but not in HIV-negative, smear-negative TB suspects in the community. However, the number needed to screen for one additional SS+ case varied widely between clinic and community screening activities.* Conclusion*. Enhanced and active TB case finding in Haiti can improve TB diagnosis and care. However, screening algorithms have to be tailored to individual settings, necessitating long-term commitment.

## 1. Introduction

Haiti has the highest tuberculosis (TB) prevalence in the Americas with 254 cases per 100,000 persons and an incidence rate of 206 cases per 100,000 persons per year according to World Health Organization (WHO) estimates [1]. The devastating earthquake of 2010 further compromised the already struggling health care system, including the destruction of the offices of the* Programme National pour la Lutte contre la Tuberculose* (PNLT), Haiti's National TB Program (subsequently referred to as NTP), and the main treatment center for multidrug resistant (MDR) TB, the Sanatorium of Sigueneau. Many persons were displaced and continue to live in poverty and under poor housing conditions in areas without access to TB diagnostic and treatment services. Case detection rates were below 70% until 2011 and just recently reached 76% in 2012 and 80% in 2013 [2]. In 2013, a total of 16,557 new TB cases were reported in Haiti. Of these new cases, 9,830 (59%) constituted smear-positive pulmonary TB (SS+ pTB), 4,732 (29%) were smear-negative pulmonary TB (SS− pTB), and 1,374 (8%) were cases of extrapulmonary TB (EPTB) [1]. However, estimates of incident annual cases range from 21,000 to greater than 31,000 [3]. Recent data suggests that the epidemic is much worse in high-risk populations [4]. Screening in an urban camp for internally displaced people (IDP) and a slum showed that the prevalence in these vulnerable populations might range from 380 to 1,350 per 100,000 persons [3].

Nationwide, there are many barriers to adequate case identification, including underfunding and understaffing of the health sector. Government spending on the health care sector is low. A large proportion of the health care in Haiti is provided by private entities, supported financially by international governmental and nongovernmental organizations [5]. TB diagnosis relies heavily on self-referral and passive case finding. There are insufficient laboratories and health facilities with the training and technology to support accurate diagnosis of tuberculosis. Weak implementation of quality assurance and monitoring and inconsistent usage of TB diagnostic algorithms compound the situation. However, within a strategy to expand TB services, national and international partners have been working since 2011 to expand laboratory capacities, including implementation of fluorescence microscopy and rapid molecular tests such as the fully automated Xpert MTB/RIF assay (subsequently referred to as Xpert) able to simultaneously detect* M. tuberculosis* and rifampin resistance within two hours.

The WHO recommends that active case finding focuses on certain at-risk populations [6]. The Stop TB Secretariat of the WHO has supported active TB case finding with the TB REACH grant program since 2010 [7–9]. The* Mache Chache* (“Go and Seek”) project was funded by Wave 3 of TB REACH and aimed to increase case detection in selected intervention sites in the capital of Haiti, Port-au-Prince. Port-au-Prince is in the Quest Department, which is the most populous department in Haiti and accounts for one-third of SS+ TB cases identified nationwide. Comparatively small in scale, the results and experiences nevertheless add to the knowledge on the impact of active case finding [10].

## 2. Methods

### 2.1. Setting

The target population (TP) of* Mache Chache* consisted of populations in four different areas of Port-au-Prince that had insufficient TB screening facilities and included an estimated 348,500 persons: (1) the catchment area around the Foyer Saint Camille (FSC) Hospital in northwest Port-au-Prince; (2) a large camp for displaced persons called “Canaan,” located a few miles from FSC with only one primary care clinic; (3) a clinic in an industrial park for unskilled workers (Centre Professionnel des Femmes Ouvrières, CPFO); (4) a highly populated area nested between Pétion-Ville and Port-au-Prince served by the Hôpital de la Communauté Haïtienne (HCH).

Prior to* Mache Chache*, FSC offered TB screening and treatment services; however, despite the presence of an HIV Care and Treatment program, TB case detection was extremely low (Table 2). Canaan and CPFO did not have any TB screening and diagnostic or treatment activities for their population of largely underserved individuals. HCH offered TB care and treatment in the settings of concomitant HIV care and had good overall case detection in the preceding years; however, services deteriorated after the earthquake of January 2010 and community outreach activities had decreased as a result.

**Figure 1:**
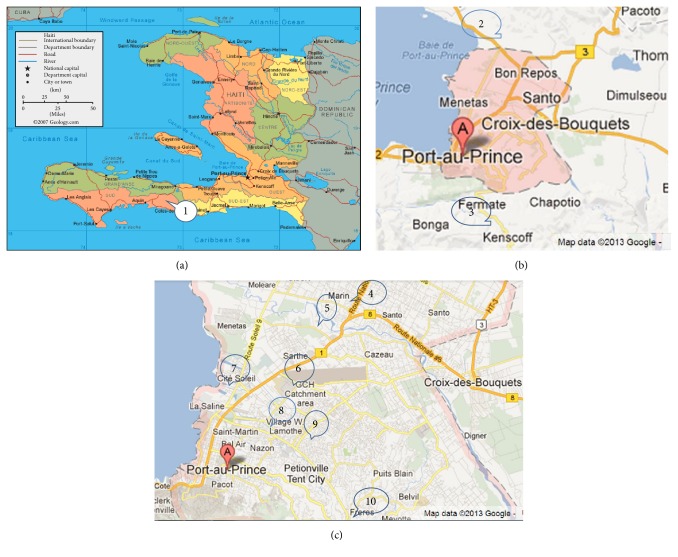
Location of the basic management units of the target population (TP), evaluation population (EP), and control population (CP). (a)* Map of Haiti*: #1: Hôpital Saint-Boniface, Fond-des-Blanc (CP). (b)* Map of Port-au-Prince (A) and surrounding area*: #2: Canaan (TP/EP). #3: Hôpital de Fermathe (CP). (c)* Map of greater Port-au-Prince (A)*: #4: Centre de Santé Lucelia Bontemps (EP). #5: Hôpital Foyer Saint Camille (TP/EP). #6: Centre Professionnel des Femmes Ouvrières (CPFO) (TP/EP). #7: CHAPI (CP). #8: Hôpital Bernard Mevs (EP). #9: Hôpital Universitaire de La Paix (EP). #10: Hôpital de la Communauté Haïtienne (TP/EP).

According to TB REACH guidelines [10], the evaluation population (EP) was defined as the TP plus nearby basic management units (BMUs) where patients screened for and diagnosed with TB could also potentially seek care. The control population (CP) consisted of populations with similar characteristics regarding health service provision and access to care which were not geographically overlapping with the EP. The final EP included an estimated 540,000 persons and consisted of BMUs at FSC, HCH and the Lucelia Bontemps Health Center, the Hôpital Bernard Mevs, and the Hôpital Universitaire de La Paix. The CP included 600,000 people and the BMUs at the health center CHAPI and the hospitals of Fermathe and Saint-Boniface in Fond-des-Blancs (see map in Figure 1). Target numbers for additional cases of SS+ and all forms of TB were estimated based on published TB prevalence rate in communities similar to the TP [3].

### 2.2. Education, Training, and Supervision

Prior to the start of the project, all laboratory technicians involved in sputum smear preparation and reading at any of the project sites participated in a five-day TB diagnostics refresher course conducted in Port-au-Prince by a laboratory specialist from the Institute of Human Virology (IHV), University of Maryland in Baltimore, Maryland. Bench aids for guidance on smear preparation, staining for acid-fast bacilly (AFB) and slide reading were provided to each laboratory.

Physicians and nurses at each site participated in didactic lectures outlining the goals of the project, as well as principles of TB screening, diagnostics, and management. A site project lead was identified and individually trained, as were the TB clinic nurses at FSC and HCH. Nurses, community health workers (CHW), and volunteers were also instructed by the technical team of the IHV office in Port-au-Prince, which included a former nurse of the NTP. Regular training and feedback sessions were held with the dedicated site project leaders and the clinic nurses. The technical team of the IHV office visited each site at least once per week and was always available to answer questions.

Clinicians, nurses, CHW, and volunteers received salary support based on their involvement in the program. At Canaan, volunteers received monthly stipends, but the support was restructured after a few months to performance-based incentives.

### 2.3. Verbal Screening and Sputum Sampling

The verbal symptom screening questions included cough of any duration, hemoptysis, fever, weight loss, and night sweats [11, 12]. A verbal screen was positive and identified a person as TB suspect if a cough of any duration was reported or, in the absence of cough, if at least two other symptoms were reported [3]. All patients presenting to the hospital and clinics at FSC, Canaan, HCH, and CPFO were screened verbally and those who tested positive were referred to sputum collection. One spot sputum was collected and patients were given two containers to collect one further spot sputum and one early morning sputum. The patients were instructed to return the additional samples the next day. CHW followed up with suspects on completion of the sputum sampling.

At FSC, HCH, and Canaan, CHW and volunteers were assigned to specific areas where they conducted daily verbal screening and contact tracing. At FSC and HCH, TB suspects were invited to come to the hospital for further screening. At Canaan, spot sputa were collected at the people's houses and CHW returned the next day for the additional samples. In addition, mobile clinics were conducted at each site. Sputum samples from Canaan were transported to FSC for microscopy with a courier at least twice per week.

Project activities were stopped at CPFO in March 2014, as the site was not able to implement technical recommendations. Project activities were partially suspended at HCH after April 2014 because of internal administrative struggles at the hospital.

### 2.4. Diagnostic Methods

All participating laboratories received new Zeiss Primo Star iLED microscopes for the project. Fluorescence microscopy testing had not been widely rolled out by the NTP yet and therefore was not performed. TB suspects were instructed to provide three sputum samples, including one early morning sample. As per NTP guidelines, TB suspects who had only one sputum sample processed were not considered screened if this sample was smear-negative. Note that mycobacterial culture is not routinely performed in Haiti.

As an additional intervention,* Mache Chache* introduced Xpert testing at FSC. The site became a member of the national Xpert network. For SS− TB suspects, an Xpert test was performed on the second or third sputum, whichever was of better quality. Samples from FSC and Canaan were processed on a continuous base; samples from HCH were stored at HCH for a maximum of 3 days and brought to FSC twice per week for processing.

A TB case was defined as bacteriologically positive if ≥1 AFB smear was positive or if the Xpert test result was positive and the patient was referred to the respective TB clinic for treatment. A physician evaluated SS−/Xpert-negative TB suspects and the diagnosis of SS− pTB or EPTB was made on clinical grounds, utilizing chest X-ray upon clinician's discretion and financial circumstances.

The diagnosis of latent TB infection (LTBI) in HIV-negative patients is based on a tuberculin skin test (TST) ≥ 10 mm in the absence of signs or symptoms of active TB. The cut-off for a positive TST in HIV-infected persons is 5 mm. Of note, universal Bacille Calmette-Guerin (BCG) vaccination at birth is recommended for all children except those born to HIV-infected mothers until HIV infection has been ruled out in the child. Prior BCG vaccination does influence diagnostic criteria for the TST.

### 2.5. External Quality Assessment (EQA) of Sputum Specimens

Fifty-one stored slides from within the previous 6 month were randomly collected from FSC in February 2014 and 100 from HCH and a blinded rechecking was performed by two experienced technicians (controllers) in Baltimore [13, 14]. Criteria used for the assessment of the smear quality can be found in Supplementary Table S1 in Supplementary Material available online at http://dx.doi.org/10.1155/2016/8020745 [15]. HCH and FSC were asked to provide all quality review reports performed by the* Laboratoire National de Santé Publique* (LNSP) during the project period, but only one report was available at HCH. LNSP criteria for slide assessment are listed in Supplementary Table S2 (personal communication).

### 2.6. TB Treatment

The TB clinic at FSC treated patients from FSC and Canaan. Patients identified at CPFO were also referred to FSC for treatment. The TB clinic at HCH cared for its own patients.

First-line TB treatment in Haiti consists of two months of isoniazid (INH), rifampin (RIF), pyrazinamide, and ethambutol, followed by a continuation phase of four months of INH and RIF. Multidrug resistant (MDR) TB cases and suspects from BMUs in Port-au-Prince are generally referred to a specialised center at the Institute of Infectious Diseases and Reproductive Health (IMIS)/Haitian Group for the Study of Kaposi's Sarcoma and Opportunistic Infections (GHESKIO) for further evaluation and treatment. Recommended treatment for LTBI consists of INH for 6 months. Of note, in the absence of active TB, HIV-infected persons are supposed to receive at least 6 months of INH treatment, independent of TST results.

### 2.7. Data Collection and Analysis

Data were collected from the NTP for each BMU for quarterly reporting to the sponsor. Of note, SS- and Xpert-positive cases were notified under smear-positive/bacteriologically positive (SS+/B+) cases per NTP guidelines. Project data were collected from each intervention site on a monthly basis by the technical team in Port-au-Prince. The teams in Haiti and in Baltimore verified data and discrepancies were addressed. Additionally, historic case notification data were collected from 2011 to 2013 from the NTP. For 2010, no verified NTP data were available and data were provided by international organizations. For 2011, historic data were not available from the NTP for FSC and data were directly collected at the site.

A retrospective chart review was conducted on 108 patients with pTB treated consecutively at the FSC TB clinic. Data were obtained from the clinical and laboratory registers and included age, gender, HIV serostatus, presence of symptoms in the verbal screen, and characteristics of the sputum as recorded in the laboratory register. Composite sputum characteristic and sputum grade scores were created that consisted of the highest respective sputum characteristic or sputum grade from the available samples from a patient. Univariate and multivariable logistic regressions were used to assess relationships between patient demographics, HIV serostatus, sputum sample characteristics, and final AFB smear-positivity positivity. All statistical calculations performed with Stata (Version 13.1, StataCorp, College Station, TX).

### 2.8. Ethical Considerations/Role of the Funding Source

TB REACH funded this project through the Stop TB Partnership. The sponsor had no influence on study, design, or data collection but was informed of the progress through routine quarterly reports. The project was approved by the NTP. All individuals with access to any patient records in the study were involved in the implementation of the project. Summary statistics were used without patient identifiers. The corresponding author had full access to all the data from the project and was responsible for undertaking the study. As the study involved the retrospective analysis of routine information from the project, individual patient consent was judged not to be required.

## 3. Results

### 3.1. Screening, Diagnosis, and Treatment Cascade

The project screened 94,867 persons for TB symptoms and identified 11,150 TB suspects (11.75%; expected: 15%). 6,812 persons (61% of TB suspects) submitted at least two sputum specimens for smear microscopy. More than 1,000 TB suspects submitted only one sputum sample and were not counted as screened per NTP guidelines if that smear was negative. Of note, with 39% overall, the dropout rate was much higher than expected and even reached 65% at Canaan (expected: hospital: 10%, community: 20%).

One hundred eighty-eighty patients (2.76% of TB suspects screened) were diagnosed with SS+ pTB and 155 with SS− pTB or EPTB. An additional 31 patients were SS− but tested Xpert-positive (total of 374 cases of all TB forms). The percentage of TB suspects diagnosed with SS+ was much lower than our estimates of around 5% of TB suspects in the community, 10% in the hospital setting, and 20% amongst Xpert tested subjects. Site- and intervention-specific results are listed in Table 1.

At FSC and HCH, 341 people were screened via household contact tracing, below the expectation of five people per TB case. One hundred fifty-three TB suspects were identified by verbal screen and examined for TB. Nine smear-negative pTB cases were identified and placed on treatment (2.6% of contacts screened, 5.9% of contacts examined). Contacts who ruled out for active TB but had a positive TST were treated for LTBI.

### 3.2. Additional NTP Notification Rates

In the 15-month intervention period (July 2013–September 2014), 361 cases of SS+/B+ cases and 688 of all TB forms were reported to the NTP in the EP compared to 261 SS+/B+ cases and 503 of all forms in the CP. The annual SS+/B+ notification rate in the EP increased from 34/100,000 to 54/100,000 persons (59% increase; 95% CI: 4–143%; *p* = 0.03). In the CP, SS+/B+ notification was 31/100,000 before intervention and 35/100,000 during the intervention (13% increase; 95% CI: −30%–83%; *p* = 0.63).

Additional 135 SS+/B+ cases were detected in the EP, representing a 60% increase from baseline but only 12% of the project target of 1066 additional SS+/B+ cases. Due to the structural issues at two intervention sites (CPFO and HCH), the project only ran at FSC and Canaan (which reported under FSC's BMU to the NTP) for the full intervention period. Comparing historical data with the intervention period at FSC, an increase from 20 SS+/B+ cases reported in the five quarters before the intervention to 124 SS+/B+ cases during the project period was noted (620% increase). Of the 124 cases, 108 (87%) were SS+ cases and 16 (13%) were SS−, Xpert-positive cases. There were 120 cases of all TB forms at FSC in the 5 quarters before and 205 during the intervention (71% increase).

In the EP, SS+/B+ cases represented 55.7% of all TB cases during the intervention compared to 37.8% before (OR 2.67; 95% CI: 1.64–2.61; *p* < 0.001); at FSC, 60% were bacteriologically confirmed during* Mache Chache* (52% SS+; 8% Xpert-positive) compared to 16.6% before (OR 7.65; 95% CI: 4.27–14.04; *p* < 0.001) (Table 2). In the CP, 55.7% were SS+ before the intervention and 56.25% during the project period. Of note, SS− and EPTB cases decreased in the EP over the intervention period and nationwide from 2013 to 2014 (Table 2).

The male-to-female ratio of SS+ cases in the EP was 1.06 and in the CP 1.02, similar to the national ratio of 1.1 [1]. In the EP, 1.5% of SS+ TB patients in the EP were younger than 15 years and 2.35% in the CP; 15.4% were 45 years or older in the EP and 19% in the CP.

### 3.3. Impact of Active Case Finding on Smear Grade

At FSC, the smear grades of all SS+ samples identified in the laboratory in the 12 months before the project were compared with the grades of all positive smears in the 15 months of the intervention. Before intervention, of 52 AFB-positive smears, none were graded as scanty, 17% as 1+, 46% as 2+, and 36% as 3+. During the intervention, of 225 AFB-positive smears, 11% of smears were graded as scanty, 31% as 1+, 7.1% as 2+, and 51% as 3+. Comparing the distribution of lower grade (scanty and 1+) and higher grade (2+ and 3+) samples between the year before and the year of* Mache Chache*, the odds of a lower sputum smear grade during the project were 3.43 (95% CI: 1.54–8.36; *p* = 0.001).

### 3.4. Xpert MTB/RIF® Screening

The Xpert screening activities only began six months into the program, after training by LNSP had been completed. Testing was initially restricted to HIV+, SS− TB suspects. During the project period, 270 HIV+, SS− TB suspects were screened and 20 were Xpert-positive (7.4%). One case of rifampicin resistance was detected and referred to IMIS. After April 2014, Xpert testing was also made available to HIV-negative, SS− TB suspects. Given the low sputum smear-positivity, Xpert testing provided an alternative diagnostic method, less affected by the technical variations of the smear described below. Furthermore, enrollment of HIV-infected patients was slower than expected. However, of 462 TB suspects with negative or unknown HIV status, only 11 (2.4%) tested positive (nine were known to be HIV-negative; HIV test results were not available for two patients). Of 751 tests performed, 7 tests were indeterminate for rifampin resistance, 24 resulted in errors, and 5 had no result.

### 3.5. Treatment Uptake

Of 219 patients diagnosed with SS+ TB, 203 (93%) started treatment; of 374 patients with all forms of TB, 346 (93%) began treatment. Of the 31 patients diagnosed by Xpert, only 23 (74%) were started on treatment and 4 patients passed away before referral. Mean number (±SD) of days between diagnosis and treatment in 19 Xpert-positive patients with available data was 4.38 ± 2.18 days. Treatment outcome data were only available for Q3 2013 and revealed that 80% of patients were cured, the same percentage as in the four quarters before the start of project.

### 3.6. Sputum Smear Quality Review/EQA

Six months into project activities, 51 smears from FSC and 100 smears from HCH underwent blinded rechecking in Baltimore. No error in examination and reporting of positive or negative slides results was found. However, the sputum smear quality was judged as poor in 71% of FSC's and 67% of HCH's samples. Most samples had greater than 10 epithelial cells/field suggestive of saliva. Only 70% of smears at FSC met size recommendations and 40% of those at HCH. Thirty-three percent and 50% of slides were too thin, respectively, and 7% and 10% were too thick; 25–34% of slides had crystals or debris and 60% of slides had poor staining quality at HCH but only 9% at FSC.

Only one quarterly review of 24 slides from HCH performed by the LNSP was available. Two slides were positive at HCH and LNSP (one 2+ at both, the second labeled 1+ at the site and 2+ by LNSP). Three slides (12.5%) reported negative by the site were found to have scanty AFB on review and classified as low false negative, minor errors. Specimen concentration was recommended. Specimen quality was found to be adequate in 83% (20/24; 4 labeled as saliva), size in all, thickness in 58% (14/24), and colorization in 88% (21/24).

Recommendations were provided to the sites on ways to improve the quality of collected sputum samples as well as staining techniques. Smear concentration could not be performed due to concerns on infection control in both laboratories.

### 3.7. Sputum Characteristic and Smear Positivity in TB Patients

**Figure 2:**
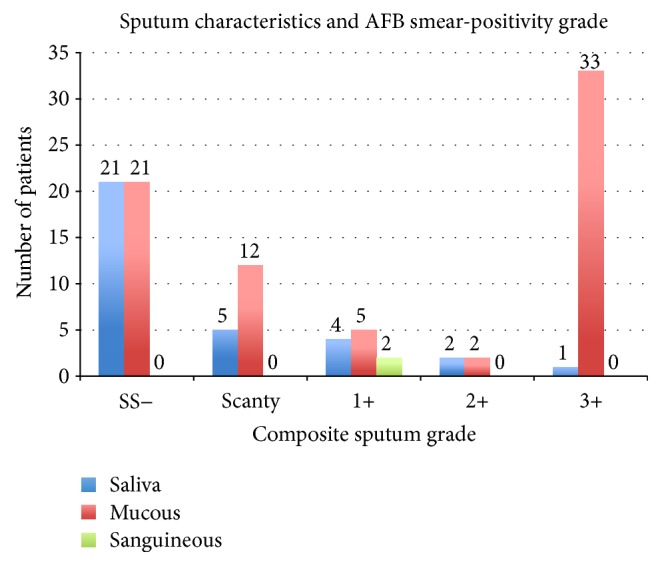
Composite sputum characteristics (saliva, mucous, and sanguineous) versus composite smear grades of 108 pulmonary TB patients at FSC. Composite sputum characteristic and sputum grade consist of the highest respective sputum characteristic or sputum score from available samples for one patient. SS−: smear-negative. The number above each column represents the number of patients in that category.

Given the problems with specimen and smear quality, a retrospective review of 108 patients with pTB was conducted at the FSC TB clinic to assess associations between sputum sample characteristics and clinical diagnosis. Patients were treated between 11/19/2013 and 08/26/2014. Demographic characteristics and results of the symptom screen and AFB smear are presented in Table 3. Mean age was 37.5 years (range 3–100). Cough was present in 72 (89%) of 81 cases with available data. Two or more of the other symptoms (fever, night sweats, weight loss, and hemoptysis) were present in 36 (60%) of 60 patients with available information. 66 patients (61%) had positive AFB smears and 33 samples (31%) were only salivary.  Figure 2 shows the composite sputum characteristics across sputum smear results and composite sputum grades. Of the 66 SS+ cases, 52 (79%) had produced at least one mucoid sample, two (3%) had produced at least one sanguineous sample, and 12 (18%) had submitted only saliva samples. Of the 33 salivary samples, 21 (64%) were SS−, and 12 (36%) were SS+. Of those, two-thirds were scanty or 1+ in sputum grade. Of the 73 mucous samples, 52 (79%) samples were SS+, of which 33 (63%) had a sputum grade of 3+. Both sanguineous samples were SS+, with a 1+ sputum grade.

In a univariate logistic regression analysis, being older than 30 years was significantly associated with having a negative smear (OR: 0.29, *p* = 0.02), as was being HIV-positive (OR: 0.19, *p* < 0.0001) (Table 2). Mucous/sanguineous samples were 4.5 times more likely to be AFB smear-positive than salivary samples (*p* = 0.001). In the multivariable analysis, age > 30 years and HIV infection remained negatively associated with AFB smear-positivity and mucous/sanguineous sputum positively (Table 4).

## 4. Discussion

We describe experiences and results of a 15-month TB case finding project called* Mache Chache* in Port-au-Prince, Haiti. Case finding activities were preceded by intensive training and retraining was provided to clinical and field project staff.

### 4.1. Impact on SS+ TB Case Notification

In the EP, SS+/B+ confirmed case notification increased by more than 50%. This was mainly due to a fivefold increase in the most active intervention site (*Hôpital Foyer Saint Camille*, FSC). At FSC as well as in the EP the proportion of SS+ cases amongst all TB forms was very low before the intervention, suggesting poor laboratory performance [3]. During* Mache Chache*, the proportion of SS+ cases increased close to the national average of 60%; to the contrary, in the CP, the proportion of SS+ cases was at the national average before the project. Based on the sensitivity of the Ziehl-Neelsen smear, SS+ cases are expected to constitute around 60% of all TB cases [3]. A lower proportion may be suggestive of poor laboratory performance; a higher one may imply underdiagnosis of clinical (SS−) TB. Unsurprisingly, there is a wide variation of the proportion of SS+ cases to all forms of TB amongst different national TB programs [16]. At FSC, the SS+ case increase was accompanied by a significant decrease of the grades of positive AFB smears, suggesting improved diagnostic performance and earlier case detection through training and active case finding in line with reports from studies in Cambodia and South Africa [17, 18].

### 4.2. Performance of the Verbal Screening Tool and Evaluation of TB Suspects

Target numbers for additional TB cases were estimated based on reported TB prevalence in comparable populations in Haiti [3, 4, 19]. However, the number of SS+/B+ cases remained well below those targets, possibly due to a combination of factors such as targets being an overestimate for the specific TP and project performance issues. The latter may include limited activities at two of the four sites, high dropout rate of TB subjects, poor sputum smear quality, and delayed start of Xpert screening. The dropout rate at most sites was much higher than expected, especially at Canaan. Contributing to this was that many patients only gave one sputum sample and were not counted as screened per NTP guidelines if that sample was smear-negative. Furthermore, the brevity of the program added to difficulties in developing a strong community program for TB case finding. This was particularly true for Canaan which did not have any infrastructure for TB diagnosis and treatment before the start of the program. Hence, community awareness for TB was low and contributed to poor follow-up from TB suspects. Financial constraints impeded the quality of the work of CHW as well as the availability of TB suspects to complete the evaluation.

An additional concern regarding the TB case detection being below targets is that the verbal screening tool, which emphasized the presence of cough, had poor specificity in this setting. The presence of cough of any duration was chosen as a sufficient single symptom for a positive verbal screen based on previous studies in Haiti that found active TB in 12–32% of coughers. Our verbal screening algorithm identified 12% of screened people as TB suspects, but the percentage of subjects diagnosed with SS+ TB was much below the estimates for most sites [3, 19]. Estimates for sensitivity and specificity of cough of any duration in TB screening have been found to range between 0.519 and 0.627 and 0.767 and 0.815, respectively, with strong dependence on the HIV prevalence [11, 20]. Unfortunately we were unable to collect summary data on the distribution of the cough and the other four symptoms across all TB suspects; however, in our review of 108 TB patients, all SS+ patients had reported cough, as did 67% of SS− patients. A dry cough was reported by many people and was more likely to be associated with a saliva specimen as compared to a productive cough (data not shown). Saliva specimens were more likely to be smear-negative, as were sputa of HIV-infected people and people above 30 years of age.

### 4.3. Impact on SS− TB Case Notification

The project included educational sessions for clinicians, emphasizing a thorough assessment of TB suspects even if smears were negative. Nevertheless, the number of SS− pTB and EPTB diagnosed during the intervention decreased in the EP (the same trend was seen nationwide in 2014). This is similar to a finding from a TB REACH project in Nepal which reported a large decrease of empiric treatment of smear-negative resulting in decreased notifications of all forms of TB in the setting of an increase of microbiologically confirmed cases through Xpert use [21]. However, opposing results were found in Cambodia, where the proportion of SS− cases increased from 40% to 70% during community-based active case finding activities [17]. Similarly, a case finding study in Pakistan revealed a larger proportional increase in SS− than SS+ cases [22]. Possible explanations for our findings include the following: (1) the number of all forms of TB identified is close to the true number and the intervention shifted cases from SS− to SS+ or (2) health care providers are hesitant to make a diagnosis of SS− TB. The project tried to address this latter issue by providing retraining to clinicians and emphasizing the national TB screening algorithms. Clinicians may not pursue a clinical diagnosis of SS− TB and trust a negative smear or Xpert result, if they know that the laboratory capacity has been upgraded through project activities. Alternatively, other providers may arrive at a clinical TB diagnosis earlier even with a negative smear, if they are sensitized to TB through an ongoing active case finding activities. Along these lines, Theron et al. have found that high rates of empirical TB treatment initiation may offset the clinical impact of improved diagnostic evaluations [23, 24]. The short duration of* Mache Chache* and the difficult infrastructure at hospitals such as HCH limited continuous supervision of clinicians. Furthermore, one of the barriers in the diagnosis of SS− TB was that X-ray availability for TB suspects was limited. Foreign donors fund most hospitals in Haiti with little direct revenue; the current cost of a (nondigital) chest X-ray in Haiti is USD 20 and would have to be paid for from the operating hospital budget or the patients as grant funding was insufficient to cover X-rays for all TB suspects and the NTP does not provide funds for radiology. TB screening algorithms, including those incorporating Xpert, are known to perform better if X-ray screening is available either for the primary or secondary screen [20, 25]. Hence it remains imperative that adequate access to affordable radiologic modalities are available to assist clinicians with TB screening algorithms without burdening the patient [26].

### 4.4. Laboratory Performance and Quality Control

An EQA of the slides at FSC and HCH found many insufficiencies in the smear quality of specimens and smear preparation without discrepancies in slide reading. These issues conceivably contributed to underdiagnosis of SS+ cases. While quarterly quality reviews by the LNSP are supposed to be scheduled for each BMU, only one written report was available during the 15-month intervention period. Less deficiencies in the quality of smears were reported, but 12.5% were found to be low false negatives which can also contribute to missed SS+ cases. It is practice in the laboratories at FSC and HCH to not reject saliva samples once they are delivered to the laboratory. The retrospective chart review revealed that 18% of SS+ TB patients had submitted only saliva specimens, similar to what has been reported from India [14]. These patients may have likely been missed if their specimens had been rejected without collection of higher quality samples. However, fieldworkers and nurses should closely supervise and instruct patients in adequate sampling. Several training and feedback sessions for CHW and nurses were held with small impact over time. Intervention programs that provide detailed instruction and supervision for sputum donation have been shown to be successful in improving the quality of sputum samples and smear positivity [27]. Our experiences emphasize that TB control programs should pay close attention to continuous EQA which should include sample quality and smear preparation. Supervision of sputum donations in patient groups with higher likelihood of providing saliva samples and having negative smears such as HIV-infected patients and patient >30 years of age should be provided and the benefit of Xpert testing evaluated. Additional research may be necessary to help improve criteria for verbal screening in Haiti and similar settings, including the optimal cut-off for the duration of cough and the quality of the cough (productive or dry).

### 4.5. Implementation of Xpert Screening

We successfully implemented Xpert screening at FSC. Haiti's national guidelines recommend Xpert screening for patients at risk for MDR-TB as well as for populations with notoriously poor AFB smear sensitivity such as HIV-infected patients and children [3]. In the current study, 7.4% of SS− HIV+ TB suspects had a positive Xpert test, supporting the use of the test for this population [28]. We expanded the inclusion criteria after 2 months with approval of the NTP to evaluate whether Xpert could improve the diagnosis in HIV-negative TB suspects. However, only 2.4% of HIV-negative TB suspects had a positive Xpert test. This is below the percentage reported from other TB REACH sponsored interventions where positivity rates reached up to 20% when all TB suspects were tested with Xpert and up to 8–13% when SS− TB suspects were tested [29]. However, in a recent study in Cambodia only 5 of 125 (4%) HIV-negative, SS− TB suspects with persistent symptoms had a positive Xpert result [30]. In our setting, a poor specificity of the verbal screen and the poor quality of many sputum specimen may have impacted Xpert testing. However, our findings would currently not support a role for testing of all SS− TB suspects with Xpert in Haiti.

### 4.6. Number Needed to Screen and Costs per SS+ Case

For our health care facility based interventions, the number needed to screen (NNS) and examine (NNE) ranged from 89 and 12 at HCH to 373 and 42 at FSC, respectively. While these numbers fall into the range described in a recent systematic review, the variation between HCH and FSC is not easily understood [31]. The same screening algorithms were used at both facilities and the slide review revealed shortcomings in both laboratories. However, wide variations between different interventions sites has been described by other TB REACH funded projects, such as Karachi, Pakistan, were the NNS for the main hospital TB clinic was 124 compared to 763 at general practitioner clinics [8]. For the community-based interventions, the NNS was >1600 and the NNE was 50 at Canaan and 61 at CPFO. For countries with medium TB incidence rates, similar results have been reported for screening activities that employed mainly symptom screening and smear testing [31]. It is likely that the relative short duration of the interventions and the lack of prior experience of staff at CPFO and Canaan contributed to suboptimal outcomes. Furthermore, the grant budget allowed for only limited salary support, especially for community volunteers. Over the course of the intervention it became evident that performance-based incentives lead to better motivation and results, as has been demonstrated elsewhere [32]. The cost per additional SS+ patients diagnosed during* Mache Chache* was 2,643 USD, an unsustainable amount, especially without external funding. However, while the TB REACH program budgets 350 USD for each projected additional SS+ case, Creswell et al. showed in a review of 28 TB REACH funded projects that an average of 864 USD were spent, with a wide spread between programs and some projects that did not find any additional SS+ case [7].

Limitations of our study include that only four intervention sites were included and that only two of them conducted screening activities for the entire time period. The project had a large drop-off between the number of TB suspects and those screened with sputum microscopy. Furthermore, individual level data on symptom screening were not available to us, neither was microbiological culture to ascertain a TB diagnosis in smear-negative TB suspects.

## 5. Conclusions

Our experiences and results suggest that retraining of health care workers and enhanced screening in health care facilities increase TB case detection and the proportion of SS+ cases, thereby conceivably contributing to earlier case detection. However, community-based screening might have a low yield in this setting, as has Xpert screening outside of the high-risk populations addressed in the Haitian national guidelines. A verbal symptom screening will have to be further evaluated in the local settings, especially for the optimal cut-off for duration of cough. External quality supervision for the laboratories and review of sputum specimens and smears are important to assess deficiencies and guides focused retraining. The proportion of SS+/microbiologically confirmed cases at a BMU may help to define intervention goals, but overall notification rates need to be carefully monitored to understand the impact of case finding and improved diagnostic tests on the clinical diagnosis of TB. Hot spots of TB transmission need to be identified to further tailor screening programs to local needs [33].

## Supplementary Material

Supplementary Table 1 displays the criteria used for the assessment of sputum quality and the grading of size, evenness, thickness, cleanness and staining quality of the smear, used by the laboratory in Baltimore. Supplementary Table 2 displays the criteria used by Haiti's National Laboratory for assessment of sputum quality and smear preparation and staining quality.

## Figures and Tables

**Table 1:** Target numbers and screening success per intervention site.

	Focus of active/enhanced case finding					Total
	Health care facility		Clinic	Community	Targeted	
	FSC	HCH	CPFO	Canaan	MTB/RIF	
Target number of people screened	12,629	24,780	8,900	50,000	1,500	97,809
Number of people actually screened	30,624	6,209	3,388	54,646	NA	94,867
Number of TB suspects	4,265	1,010	260	4,883	732	11,150
Number of TB suspects examined for TB	3,449	806	121	1,704	732 (270^*∗*^)	6,812
Number of SS+/B+ TB cases	82	70	2	34	31 (20^*∗*^)	219
Number of SS+/B+ TB cases started on treatment	74	72	2	32	23	203
Number of all forms TB cases	201	103	2	37	NA	374
Number of all forms TB cases started on treatment	190	97	1	35	NA	346
% of people screened identified as TB suspects	14%	16%	8%	9%	NA	12%
% of TB suspects examined	81%	80%	47%	35%	NA	61%
% of people screened diagnosed with SS+/B+ TB	0.3%	1.1%	0.1%	0.1%	NA	0.2%
% of TB suspects examined diagnosed with SS+/B+ TB	2.4%	8.7%	1.7%	2.0%	4% (7.4%^*∗*^)	3%
NN screen for 1 SS+/B+ case	373	89	1694	1607	NA	433
NN exam for 1 SS+/B+ case	42	12	61	50	24	31

SS+: smear-positive; B+: bacteriologically positive (smear and Xpert MTB/RIF); NN: number needed to (Screen/Exam); FSC: Hôpital Foyer Saint Camille; HCH: Hôpital de la Communauté Haïtienne; and CPFO: Centre Professionnel des Femmes Ouvrières.

^*∗*^Data for HIV-infected subjects only.

**Table 2:** Numbers and ratios of new smear-/bacteriologically positive, smear-negative pulmonary TB and extrapulmonary TB reported to NTP in a five-quarter period prior to and during the project intervention.

Population	SS+/B+ TB (*n*)	SS− pTB (*n*)	EPTB (*n*)	pTB: ratio of SS+/B+ to SS−	Ratio of SS+/B+ pTB to all TB forms	SS+/B+: % change historic to intervention
EP historic	226	215	157	1.05	0.38	
EP intervention	361	183	104	1.97	0.56	60

CP historic	232	103	81	2.25	0.56	
CP intervention	261	132	71	1.98	0.56	12.5

FSC historic	20	56	44	0.36	0.17	
FSC intervention^**∗**^	124	63	18	1.97	0.61	620

Haiti (2013)^**∗∗**^	9830	4732	1995	2.08	0.59	
Haiti (2014)^**∗∗**^	9747	3521	1541	2.77	0.65	

SS+: smear-positive; B+: bacteriologically positive (smear and Xpert MTB/RIF); and SS−: smear-negative.

pTB: pulmonary TB; EPTB: extrapulmonary TB; EP: evaluation population; CP: control population, TP: target population; and FSC: Hôpital Foyer Saint Camille.

^*∗*^As FSC and Canaan were the only sites participating for the entire 5 quarters, only data for the FSC BMU which includes Canaan are included for the TP.

^*∗∗*^Nationwide data for four quarters of 2013 and 2014 data are from [1].

**Table 3:** Demographics and sputum characteristics of 108 patients with pulmonary TB.

	Smear+	Smear−
	*n*/*N* (%)	*n*/*N* (%)
*Patient demographics*		
Male	30/66 (45)	20/44 (45)
Age > 30 years	25/59 (42)	32/44 (73)
HIV infection	15/65 (23)	26/43 (60)

*Verbal screening results*		
Cough	52/52 (100)	20/30 (67)
Fever	24/33 (73)	14/27 (52)
Hemoptysis	5/33 (15)	6/27 (22)
Weight loss	22/33 (67)	20/27 (75)
Night sweats	9/33 (27)	10/27 (37)
2 symptoms (excluding cough)	20/33 (61)	16/27 (59)

*Composite sputum characteristics*		
Saliva	12/66 (18)	21/42 (50)
Mucous	52/66 (79)	21/42 (50)
Sanguineous	2/66 (3)	0

*Composite smear grade*		*NA*
Scanty	17/66 (26)	
1+	11/66 (17)	
2+	4/66 (6)	
3+	34/66 (52)	

*N*: number of subjects with available data; *n*: number of subjects with characteristic.

**Table 4:** Logistic regression of factors associated with AFB smear-positivity.

Patient variable	Univariate regression				Multivariable regression^*∗*^			
	*N*	OR	95% CI	*p* value	*N*	OR	95% CI	*p* value
Age > 30 years	101	0.29	0.13, 0.69	**0.005**	99	0.30	0.11, 0.83	**0.021**
Female gender	108	0.97	0.45, 2.10	0.93		1.21	0.45, 3.21	0.71
HIV infection	106	0.19	0.08, 0.45	**<0.0001**		0.26	0.10, 0.70	**0.008**
Mucous/sanguineous sputum	106	4.50	1.89, 10.74	**0.001**		6.75	2.25, 20.25	**0.001**

^*∗*^Adjusted for age, gender, HIV serostatus, and sputum characteristics.
