# Supplementary material for: Active Tuberculosis Case Finding in Port-au-Prince, Haiti: Experiences, Results, and Implications for Tuberculosis Control Programs
Source: Tuberc Res Treat. 2016 Sep 5;2016:8020745. doi: 10.1155/2016/8020745 (PMC5030475; doi:10.1155/2016/8020745)
Supplement: Supplementary file 1 — Supplementary Table 1 displays the criteria used for the assessment of sputum quality and the grading of size, evenness, thickness, cleanness and staining quality of the smear, used by the laboratory in Baltimore. Supplementary Table 2 displays the criteria used by Haiti's National Laboratory for assessment of sputum quality and smear preparation and staining quality. [file 8020745.f1.pdf]

Supplementary Table S1: Sputum quality assessment and grading system used at the Baltimore Laboratory [13-15]

Properties	Grading	
	Good	Poor
1. Sputum Quality	Presences of >25 leucocytes/field at 100X	Absence or >5 of leucocytes in the smear.<br>Presence of more than 10 epithelial cells/field
2. Size	1-2cm X 2-3cm<br>Oval in shape and smear centrally placed in the slide	Smear size smaller than 1-2cm X 2-3cm and larger than 1-2cm X 2-3cm<br>Irregular shape. Smear placed not centrally
3. Evenness	Sputum spread evenly on the glass slide not too thick not too thin	Smear too thick or too thin
4. Thickness	Mono-layer smear on the glass slide<br>Whole depth of the smear layer can be focused sharply in each field	Smear too thick with over-lapping cells seen in entire smear.<br>Smear too thin<br>Whole depth of the smear layer can be focused sharply in less than 50% of the field
5. Cleanness	Clean smear. Smear free from stain deposit, crystals produced by overheating of stain and poor maintenance of dyes. Absence of debris.	Dirty smear, cannot screen the smear on the slide.<br>Smear found with stain deposits, crystals and debris.
6. Staining quality by microscopic observation	AFB and background clearly distinguished.<br>AFB bright red in color.<br>Leucocytes stained blue and absence of stain particles.	Not able to distinguish AFB and background.<br>AFB faintly red in color.<br>Fuchsin color remaining on the background<br>Methylene blue color remaining on the un-stained parts.

Supplementary Table S2: Sputum quality assessment at Haiti's National Laboratory (LNSP)

Properties	Assessment
1. Sputum Quality	Presence of epithelial cells and white cells
2. Size	2x1 cm in the center of the slide
3. Thickness	Able to read a printed text through the dry smear
4. Color	AFB bright red on a blue background
